# Detection of lipoarabinomannan (LAM) in urine is an independent predictor of mortality risk in patients receiving treatment for HIV-associated tuberculosis in sub-Saharan Africa: a systematic review and meta-analysis

**DOI:** 10.1186/s12916-016-0603-9

**Published:** 2016-03-23

**Authors:** Ankur Gupta-Wright, Jurgens A. Peters, Clare Flach, Stephen D. Lawn

**Affiliations:** Department of Clinical Research, Faculty of Infectious and Tropical Diseases, London School of Hygiene and Tropical Medicine, London, UK; Malawi-Liverpool-Wellcome Trust Clinical Research Program, College of Medicine, University of Malawi, Blantyre, Malawi; Department of Infectious Disease Epidemiology, Faculty of Epidemiology and Population Health, London School of Hygiene and Tropical Medicine, London, UK; The Desmond Tutu HIV Centre, Institute of Infectious Disease and Molecular Medicine, Faculty of Health Sciences, University of Cape Town, Cape Town, South Africa

**Keywords:** HIV, Tuberculosis, Lipoarabinomannan, LAM, Mortality, Systematic review

## Abstract

**Background:**

Simple immune capture assays that detect mycobacterial lipoarabinomannan (LAM) antigen in urine are promising new tools for the diagnosis of HIV-associated tuberculosis (HIV-TB). In addition, however, recent prospective cohort studies of patients with HIV-TB have demonstrated associations between LAM in the urine and increased mortality risk during TB treatment, indicating an additional utility of urinary LAM as a prognostic marker. We conducted a systematic review and meta-analysis to summarise the evidence concerning the strength of this relationship in adults with HIV-TB in sub-Saharan Africa, thereby quantifying the assay’s prognostic value.

**Methods:**

We searched MEDLINE and Embase databases using comprehensive search terms for ‘HIV’, ‘TB’, ‘LAM’ and ‘sub-Saharan Africa’. Identified studies were reviewed and selected according to predefined criteria.

**Results:**

We identified 10 studies eligible for inclusion in this systematic review, reporting on a total of 1172 HIV-TB cases. Of these, 512 patients (44 %) tested positive for urinary LAM. After a variable duration of follow-up of between 2 and 6 months, overall case fatality rates among HIV-TB cases varied between 7 % and 53 %. Pooled summary estimates generated by random-effects meta-analysis showed a two-fold increased risk of mortality for urinary LAM-positive HIV-TB cases compared to urinary LAM-negative HIV-TB cases (relative risk 2.3, 95 % confidence interval 1.6–3.1). Some heterogeneity was explained by study setting and patient population in sub-group analyses. Five studies also reported multivariable analyses of risk factors for mortality, and pooled summary estimates demonstrated over two-fold increased mortality risk (odds ratio 2.5, 95 % confidence interval 1.4–4.5) among urinary LAM-positive HIV-TB cases, even after adjustment for other risk factors for mortality, including CD4 cell count.

**Conclusions:**

We have demonstrated that detectable LAM in urine is associated with increased risk of mortality during TB treatment, and that this relationship remains after adjusting for other risk factors for mortality. This may simply be due to a positive test for urinary LAM serving as a marker of higher mycobacterial load and greater disease dissemination and severity. Alternatively, LAM antigen may directly compromise host immune responses through its known immunomodulatory effects. Detectable LAM in the urine is an independent risk factor for mortality among patients receiving treatment for HIV-TB. Further research is warranted to elucidate the underlying mechanisms and to determine whether this vulnerable patient population may benefit from adjunctive interventions.

**Electronic supplementary material:**

The online version of this article (doi:10.1186/s12916-016-0603-9) contains supplementary material, which is available to authorized users.

## Background

Tuberculosis (TB) remains the most frequent cause of HIV/AIDS-related deaths globally, accounting for 0.4 million deaths in 2014 alone [1]. Diagnosis of HIV-associated TB (HIV-TB) remains challenging due to non-specific clinical features, early dissemination beyond the lungs, the relatively low mycobacterial burden within sputum samples, and clinical over-reliance on sputum-based diagnostic tests [2–4]. Mycobacterial culture is still regarded as the ‘gold standard’ diagnostic test; however, in practice its use and utility are greatly limited by prolonged turnaround times and lack of widespread availability due to the need for expensive infrastructure and skilled laboratory personnel. Diagnostic tools that are rapid, have good diagnostic accuracy and can be used at all levels of the healthcare system will be required to meet ambitious World Health Organization (WHO) goals of reducing TB deaths by 95 % and new cases by 90 % by 2035 [5–7].

Recent years have seen increased investment and research into rapid, ‘point-of-care’ diagnostics. Given the challenges of obtaining sputum samples and limited yield in extrapulmonary TB, urine has been identified as a favourable alternative biological sample due to the ease of obtaining samples from patients, the ease of laboratory handling and processing, and the lower risk of nosocomial transmission to healthcare and laboratory workers. Several mycobacterial antigens have been identified in the urine of patients with active TB [8, 9]. Promising diagnostic assays to emerge are those that detect the mycobacterial cell wall lipopolysaccharide lipoarabinomannan (LAM) using simple immune capture assays [10]. Testing for LAM in the urine has proved particularly useful in those with HIV-TB, with incrementally greater sensitivity with the progression of immunodeficiency [11–13]. In addition to commercially available enzyme-linked immunosorbent assays (ELISA), a simple, low-cost and rapid lateral flow point-of-care assay has also been developed (Determine TB-LAM; Alere Inc., Waltham, MA, USA). This is undergoing impact evaluation as part of the diagnostic algorithms for HIV-TB in clinical trials in sub-Saharan Africa [14–16], and WHO have conditionally recommended its use to assist in TB diagnosis in hospitalised patients with low (≤100 cells/μl) CD4 cell counts or patients who are seriously ill [17].

The diagnostic accuracy of urinary LAM detection for HIV-TB has been extensively studied and is the subject of a comprehensive Cochrane systematic review and meta-analysis [18]. However, recent prospective studies of diagnostic accuracy have also highlighted its prognostic value, demonstrating strong associations between the detection of urinary LAM and mortality risk during follow-up on TB treatment; this association persists even after adjustment for key confounding factors such as blood CD4 count, blood haemoglobin level and age [19]. Testing for LAM in the urine may therefore be of additional clinical benefit, over and above the diagnosis of TB, by identifying patients with the highest mortality risk who may potentially benefit from closer follow-up or adjunctive interventions used in combination with TB treatment, anti-retroviral therapy (ART) and co-trimoxazole prophylaxis.

We have performed a systematic review and meta-analysis to summarise the strength of the relationship between urinary LAM and mortality in adults with HIV-TB. We also discuss potential mechanisms underlying these associations and discuss the need for future research and implications for the implementation of testing for urinary LAM in this vulnerable population.

## Methods

### Search strategy

We searched MEDLINE and Embase databases for studies reporting urinary LAM status in HIV-infected adults, and published up until 1 November 2015. The search strategy involved combining four search ‘sets’ with the Boolean operator ‘AND’. The search sets included comprehensive terms for ‘tuberculosis’, ‘HIV/AIDS’ and ‘lipoarabinomannan’, and ‘sub-Saharan Africa’. References of relevant studies and review articles were also searched, and experts in the field contacted to suggest additional references. The search strategy was pre-specified in the review protocol (Additional file 1: Table S1). In addition, abstract books from the International Union Against Tuberculosis and Lung Disease were manually searched, and abstracts from the Conference of Retroviruses and Opportunistic Infections were electronically searched (both from 2007 to 2015). The studies identified were compiled into a database and screened on title and/or abstract, with duplicates removed. Full texts of those potentially eligible articles were reviewed further. This review was conducted and reported in accordance with the PRISMA checklist [20]. Research ethics permission was not sought because this was a secondary analysis of published anonymised data.

### Study selection

Identified studies were included if they reported mortality and urinary LAM status in adult patients with HIV-TB co-infection, if they had at least 10 TB cases with urinary LAM results and at least five deaths, and if they were undertaken in sub-Saharan Africa. Studies were excluded if they did not report mortality outcomes (our primary outcome of interest), if they related only to paediatric populations, or if they were studies of particular sub-populations that were not easily generalisable (e.g., miners or prisoners). Studies including both HIV-positive and HIV-negative patients with TB were only included if they presented disaggregated data based upon HIV status or if <10 % of the patients with TB were HIV negative. Non-English-language studies were only included if adequate data could be extracted from an English abstract.

### Data extraction and analysis

Data were extracted directly into a database by two reviewers, including study citation, year of publication, setting (country/healthcare level), number of patients and TB diagnoses, baseline characteristics, TB reference standard, method of testing for urinary LAM, number of LAM-positive and LAM-negative TB cases, mortality in patients diagnosed with TB (overall and stratified by urinary LAM status), and risk factors for mortality. The primary outcome was the risk of mortality in urinary LAM-positive TB cases compared to LAM-negative TB cases. Case fatality rates were calculated based on total number of TB cases with follow-up data and total number of deaths. The other outcome of interest was adjusted odds ratio (OR) of mortality for urinary LAM-positive TB cases (based on a multivariable analysis including other predictors of mortality). Studies were included in the analysis if these data were presented. If adjusted OR of death was not presented, the author was contacted to ask if those data were available. Study quality was graded according to a pre-specified checklist, which was adapted from the QUADAS-2 tool (see Additional file 1: Table S2) [21].

All analyses was done using Stata 11.0 (StataCorp, College Station, TX, USA) and were done on study-level data. Forest plots were generated for mortality risk ratio. The heterogeneity of study outcomes were calculated using the I^2^ statistic. Pooled estimates were calculated using random-effects modelling, with study weights assigned based on inverse variance. The source of heterogeneity was explored using sub-group analyses (study setting, median CD4 cell count, overall TB mortality, time at which mortality outcome was measured). A fixed continuity correction of 0.5 was used for studies with 0 or 100 % mortality in any group. The adjusted ORs for mortality from multivariate regression analyses were also presented in a forest plot and summary statistics calculated as above. Funnel plots of log ORs against their standard error and Egger’s test were used to aid assessment of bias.

## Results

**Fig. 1:**
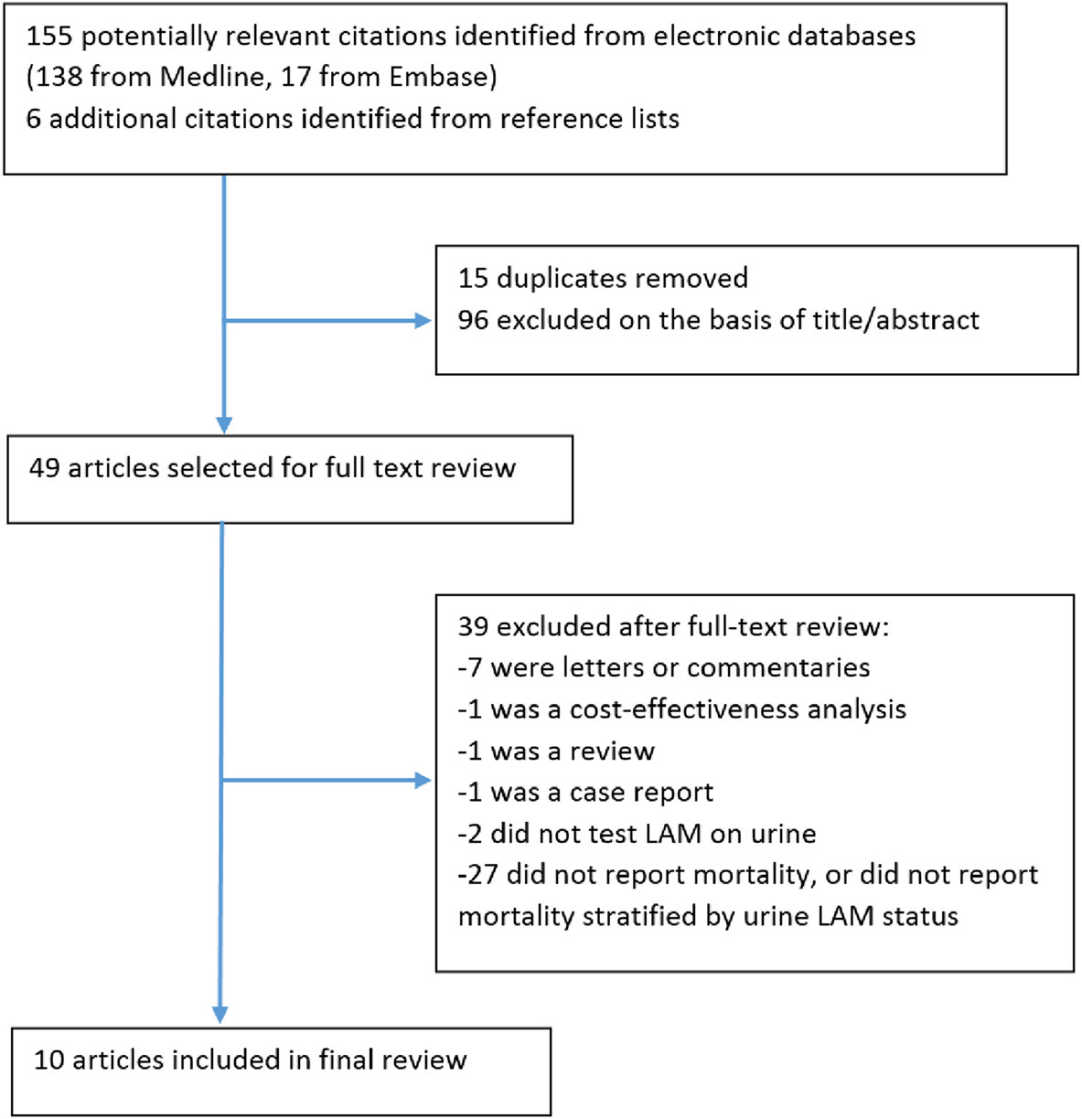
Flow chart showing study selection process. *LAM* lipoarabinomannan

A total of 161 citations were identified, 49 studies selected for full-text review, and 10 studies eligible for inclusion (Fig. 1). Included studies are summarized in Table 1. All studies were cohort in design, conducted in sub-Saharan Africa, enrolled adults ≥18 years of age, and reported between 2009 and 2015. Three studies were based in an outpatient setting, six were of hospital inpatients, and one enrolled patients from both settings. Most studies enrolled patients in whom TB was the suspected diagnosis based on clinical presentation, although three studies were done in patients due to start ART, and one study was restricted to patients with a TB diagnosis (Table 1). Two studies reported results in HIV-positive and HIV-negative patients. These were eligible for inclusion in this review because the number of HIV-negative cases comprised <10 % of the total TB cases. Eight studies used the Determine TB-LAM lateral flow assay to test for urinary LAM, and two studies used the Clearview TB ELISA (Inverness Medical Innovations, UK). All studies used standard first-line TB treatment and initiated ART in accordance with national guidelines at the time. This review includes data from 1172 TB cases and 512 (44 %) urinary LAM-positive cases in total. Five studies were deemed as being of good quality, and five of moderate quality (Table 1).

**Table 1:** Studies reporting urinary LAM detection and mortality included in the systematic review

Study	LAM assay used (type of urine sample)	Study setting and population (country)	Median CD4 cell count (by LAM status if presented) (cells/μl)	Number of TB cases/total number in study (prevalence %)	Number of urinary LAM-positive TB cases (%)	Duration of follow-up (months)	Overall mortality in TB cases (%)	Number of TB deaths/number of TB cases (%)		RR of mortality^a^ (95 % CI)	Quality assessment score^b^
								LAM positive	LAM negative		
Shah et al. (2009) [47]	Clearview TB ELISA (frozen urine)	Hospitalised patients; TB suspected (South Africa)	79	193/499 (38.7)	114 (59.1)	2	22.3	31/114 (27.2)	12/79 (15.2)	1.8 (1.0–3.3)	60
Lawn et al. (2012) [48]	Determine TB-LAM lateral flow assay (frozen urine)	Outpatient clinic; patients initiating ART (South Africa)	100 (LAM-positive 37; LAM-negative 115)	59/325 (18.2)	23 (39.0)	3	8.5	5/23 (21.7)	0/36 (0.0)	NA	70
Talbot et al. (2012) [49]	Clearview TB ELISA (fresh and frozen urine)	Hospitalised patients; TB suspected (Tanzania)	86	69/212 (32.5)	45 (65.2)	2	52.9	25/38 (65.8)	33/83 (39.8)	1.7 (1.2–2.3)	75
Peter et al. (2013) [50]	Determine TB-LAM lateral flow assay (frozen urine)	Hospitalised patients; TB suspected (South Africa)	89 (LAM-positive 62; LAM-negative 180)	116/281 (4.2)	58 (50.0)	2	13.9	6/25 (24.0)^c^	1/23 (8.5)^c^	5.5 (0.7–42.4)	80
Balcha et al. (2014) [51]	Determine TB-LAM lateral flow assay (frozen urine)	Outpatient clinic; ART naïve; sputum producers (Ethiopia)	176 (LAM-positive 94; LAM-negative 187)	128/757 (16.9)	35 (27.3)	6	6.8	7/35 (20.0)	3/113 (2.7)	7.5 (2.1–27.6)	60
Manabe et al. (2014) [28]	Determine TB-LAM lateral flow assay (fresh urine)	Hospitalised patients; TB suspects (Uganda)	57	145/351 (41.3)	90 (62.1)	2	22.1	25/90 (27.8)	7/37 (12.7)	2.2 (1.0–4.7)	75
Drain et al. (2015) [52]	Determine TB-LAM lateral flow assay (frozen urine)	Outpatient clinic; patients initiating TB treatment (South Africa)	168 (LAM-positive 106; LAM-negative 198)	90/90 (100.0)	29 (22.2)	36	27.8	9/29 (31.0)	16/61 (26.2)	1.2 (0.6–2.4)	70
Peter et al. (2015) [53]	Determine TB-LAM lateral flow assay (frozen urine)	Hospitalised patients; TB suspected (South Africa)	210	181/583 (31.0)	41 (22.7)	6	13.0	6/17 (35.2)	15/106 (14.2)	2.5 (1.1–5.5)	85
Lawn et al. (2015) [54]	Determine TB-LAM lateral flow assay (frozen urine)	Hospitalised patients; all HIV+ patients (South Africa)	148	136/427 (31.2)	53 (39.0)	3	13.7	13/5 (24.5)	6/83 (7.2)	3.4 (1.4–8.4)	75
Bjerrum et al. (2015) [55]	Determine TB-LAM lateral flow assay (fresh urine)	Hospital inpatient and outpatient; TB suspected (Ghana)	127	55/469 (11.7)	24 (43.6)	6	32.7	13/24 (54.2)	5/31 (16.1)	3.4 (1.4–8.1)	70

*ART* antiretroviral therapy, *CI* confidence interval, *ELISA* enzyme-linked immunosorbent assay, *LAM* lipoarabinomannan, *NA* not applicable, *RR* relative risk, *TB* tuberculosis. ^a^Urinary LAM-positive TB cases compared to urinary LAM-negative TB cases. ^b^Quality assessment score graded as follows: (<50 poor, 50–74 moderate, >74 good. ^c^Mortality in TB cases with urinary LAM results only reported in patients who did not receive ‘early empirical TB therapy’

The proportion of urinary LAM-positive TB cases varied from 22 % to 65 %, and was higher in studies of hospital inpatients than those of outpatients (mean 49 % and 30 % respectively, *P* = 0.04). Nine of the studies used microbiological definitions of TB cases, which included mycobacterial culture and/or the Xpert MTB/RIF assay (Cepheid, Sunnyvale, CA, USA). Three studies included only sputum samples in the reference standard, and six included at least one non-respiratory sample within the reference standard for TB diagnosis. Of the eight studies using the TB-LAM assay, four used the grade 2 cut-off as a ‘positive’ result, two used a grade 1 or higher cut-off, and two did not report which cut-off was used (Additional file 1: Table S3). Median loss to follow-up was 13.4 % (range 0–43 %, Additional file 1: Table S3).

Mortality outcomes in TB cases were assessed at between 2 and 3 months of follow-up for six studies, at 6 months in three studies, and at 36 months in one study. Overall case fatality rates among HIV-TB cases varied between 7 % and 53 %. Median CD4 cell counts varied between 57 and 210 cell/mm^3^ (overall median 114 cells/mm^3^), indicating severely immunocompromised patient populations. Loss to follow-up rates varied greatly between studies (Additional file 1: Table S3).

**Fig. 2:**
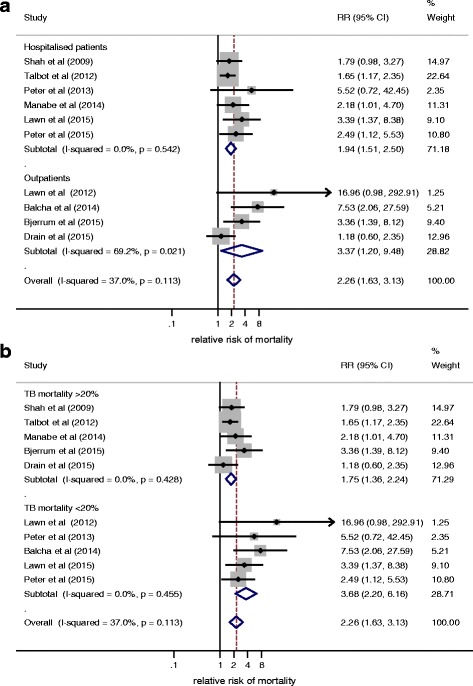
Forest plot showing relative risk (*RR*) of mortality in urinary lipoarabinomannan (*LAM*)-positive tuberculosis (*TB*) cases compared to urinary LAM-negative TB cases, stratified by (**a**) study setting and (**b**) overall mortality in patients with TB

All studies demonstrated an increased risk of mortality amongst urinary LAM-positive TB cases compared to LAM-negative cases, with the relative risk (RR) of mortality varying from 1.2 to 7.5 (median 2.5, interquartile rate 1.8–3.4; Table 1, Fig. 2). A pooled summary estimate generated using a random-effects meta-analysis showed a two-fold increased risk of mortality (RR 2.3, 95 % confidence interval [CI] 1.6–3.1; Fig. 2), but demonstrated moderate heterogeneity (I^2^ = 37.0 %, *P* = 0.113).

Sub-group analyses were undertaken to try to explain the heterogeneity in mortality risk across studies (Additional file 1: Table S4). Stratifying studies by healthcare setting removed heterogeneity amongst studies conducted on hospitalised patients, giving a pooled summary RR for mortality of 1.9 (95 % CI 1.5–2.5; Fig. 2). The summary RR of mortality was higher at 3.4 (95 % CI 1.2–9.5, *n* = 4) for outpatient studies, but showed greater variation (I^2^ = 69.2 %). A similar effect was seen in sub-group analysis by overall TB mortality, with an almost two-fold increase in mortality risk associated with LAM-positivity when overall mortality was >20 % (RR 1.8, 95 % CI 1.4–2.2, *n* = 5). Studies with overall mortality risk ≤20 % showed an even greater RR of mortality for LAM-positive patients (RR 3.7, 95 % CI 2.2–6.2, *n* = 5). Studies with a median CD4 cell count >100 cells/μl had a higher RR for mortality in LAM-positive patients than those with medians ≤100 cells/μl (RR 2.7, 95 % CI 1.5–4.7, *n* = 5 and RR 1.9, 95 % CI 1.4–2.6, *n* = 5 respectively). The summary RRs were similar when studies were stratified by time at which mortality was measured (≤3 months RR 2.1, 95 % CI 1.5–2.9, *n* = 6; >3 months RR 2.6, 95 % CI 1.3–5.2).

Sensitivity analyses showed that excluding the two studies reporting a small number of HIV-negative cases (<1 % of total TB cases included in the meta-analysis) did not alter the overall effect size. Further sensitivity analyses excluding studies using a (pre-January 2014) grade 1 cut-off for TB-LAM or not reporting cut-off grade, studies using the Clearview TB-ELISA and not the TB-LAM assay, studies with only respiratory samples in the TB reference standard, studies of low or moderate quality, and studies with >20 % loss to follow-up also resulted in no substantial change in the overall effect size. Analyses with little heterogeneity were also repeated using fixed-effects meta-analysis, which did not alter the effect size (data not shown). A funnel plot showed few studies with small or no effect size, which may suggest publication bias (Egger’s test *P* = 0.025; Additional file 1: Figure S1).

**Fig. 3:**
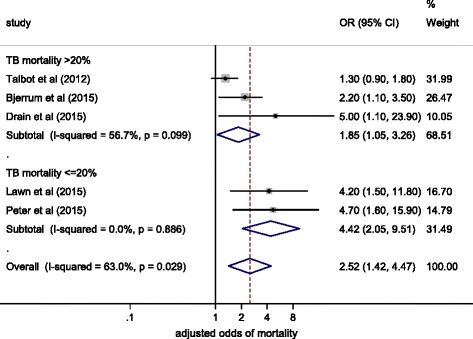
Forest plot showing adjusted odds ratio of mortality in urinary lipoarabinomannan (*LAM*)-positive tuberculosis (*TB*) cases compared to urinary LAM-negative TB cases, stratified by overall mortality in TB cases. *CI* confidence interval, *OR* odds ratio

Five studies reported results of multivariable regression analyses for mortality, including urinary LAM detection as a variable. No studies were powered to detect a difference in mortality by urinary LAM status, but three studies demonstrated urinary LAM was an independent predictor for mortality (adjusted OR varied from 2.2 to 4.7). Risk factors included in multivariable models are outlined in Table 2 and included CD4 cell count (as a marker of HIV-associated immunosuppression) in four studies. A pooled summary estimate calculated using meta-analysis showed an over two-fold increased odds of mortality (OR 2.5, 95 % CI 1.4–4.5; Fig. 3) among HIV-TB cases with positive urinary LAM tests, even after adjustment for other risk factors for mortality. However, these studies did show a large degree of heterogeneity in their effect sizes. Studies in which overall mortality was ≤20 % had a greater summary RR for mortality in LAM-positive patients (RR 4.4, 95 % CI 2.1–9.5) compared to studies with a mortality >20 % (1.9, 95 % CI 1.1–3.3; Fig. 3), although there were few studies in each sub-group and CIs overlapped.

**Table 2:** Studies reporting adjusted odds ratios for mortality in urinary LAM-positive compared to urinary LAM-negative TB cases, after adjustment for other predictors of mortality

Study	Study setting and population	Adjusted odds ratio for mortality in urinary LAM-positive compared to urinary LAM-negative TB cases (95 % CI)	Variables included in the multivariable analysis with urinary LAM status
Talbot et al. (2012) [49]	Hospital inpatients; TB suspected	1.3 (0.9–1.8)	CD4 cell count, ART
Drain et al. (2015) [52]	Outpatients; confirmed TB patients	5 (1.1–23.9)	CD4 cell count, age, gender, Karnofsky score
Peter et al. (2015) [53]	Hospital inpatients; TB suspected	4.7 (1.6–15.9)	Study site, gender, age, CD4 cell count
Lawn et al. (2015) [54]	Hospital inpatients; All HIV+ patients	4.2 (1.5–11.8)	Age, CD4 cell count
Bjerrum et al. (2015) [55]	Hospital inpatients and outpatients; TB suspected	2.2 (1.1–3.5)	Gender, hospitalisation, CD4 cell count, Medical Early Warning Score

*ART* antiretroviral therapy, *CI* confidence interval, *LAM* lipoarabinomannan, *TB* tuberculosis

## Discussion

This is the first systematic review and meta-analysis to assess the association between urinary LAM detection and mortality during TB treatment in patients with HIV-TB. The meta-analysis demonstrated a two-fold greater mortality risk among those patients with urinary LAM-positive compared to urinary LAM-negative TB. Five studies reported adjusted OR of mortality in LAM-positive TB cases, and demonstrated that urinary LAM is an independent predictor of mortality in TB cases, even after adjustment for other important risk factors for mortality, such as degree of immunosuppression. There are multiple potential mechanisms that may underlie this association (Table 3).

**Table 3:** Potential mechanisms of the association between urinary LAM detection and increased mortality risk

Potential mechanism	Evidence	References
Urinary LAM is a marker of disseminated TB and higher mycobacterial burden, which is associated with a worse prognosis	• Urinary LAM is due to haematogenously disseminated renal TB	Cox et al. 2015 [26]
	• HIV-TB patients with mycobacteraemia have a higher mortality	Cummings et al. 2015 [56]
	• Higher concentrations of urinary LAM are associated with higher mycobacterial burden	Kerkhoff et al. 2014 [19]
Urinary LAM is a proxy for a low CD4 cell count	• HIV-TB patients with positive urinary LAM tests have lower CD4 cell counts	Minion et al. 2011 [11]
	• Mortality is higher in patients with lower CD4 cell counts	Gupta et al. 2015 [34]
LAM itself contributes to immunosuppression, impairing host defences against *MTB* and other opportunistic infections	• LAM is a virulence factor for *MTB*	Strohmeier et al. 1999 [35]
	• LAM inhibits immune responses, with direct inhibitory effects on macrophage activation and function	Mishra et al. 2011 [38] Neyrolles et al. 2011 [41]
	• LAM inhibits pro-inflammatory cytokines, e.g. IL-12 and TNF-α	
	• LAM enhances the secretion of anti-inflammatory cytokines, e.g. IL-10	

*LAM* lipoarabinomannan, MTB *Mycobacterium tuberculosis*, *TB* tuberculosis

The strengths of this systematic review and meta-analysis include the synthesis of data from a large number of patients enrolled in studies in diverse settings across sub-Saharan Africa, with data from 1172 patients with TB, of whom 44 % had LAM detected in their urine. The selection of patients was well described in all studies, and was mostly representative of the population for which urinary LAM testing has been conditionally recommended in recent WHO policy guidance (patients suspected of having TB, HIV-positive patients with low CD4 cell counts, or seriously unwell patients such as those requiring hospitalisation) [17]. Studies were not powered to demonstrate a mortality difference based on urinary LAM detection, but the meta-analysis has demonstrated a robust association.

Although there effect size varied across studies, heterogeneity appeared to be moderate at most (I^2^ = 39 %). The most likely sources of variation were in the settings and patient populations. Studies conducted in hospital settings, which had higher overall TB mortality and lower median CD4 cell counts, showed approximately double the mortality risk in LAM-positive compared to LAM-negative TB patients. Surprisingly, the association between LAM-positivity and mortality was even stronger in outpatient settings, with lower overall TB mortality and higher median CD4 cell counts, although effect sizes were more variable. Urinary LAM testing may be an even better marker of poor prognosis in these settings, although recent WHO recommendations do not specify its use as diagnostic for TB in these patient populations [17].

Limitations of this systematic review include the over-representation of South Africa amongst the study settings, which is true for HIV-TB research as a whole. Different methods of detecting LAM in urine were used, as were different cut-offs for the Determine TB-LAM lateral flow assay. In addition, although most studies used mycobacterial culture from sputum as the reference standard for diagnosing TB, there was some variation in case definitions for HIV-TB. Although two eligible studies included a very small number of HIV-negative cases, their exclusion in a sensitivity analysis did not impact the overall effect size. All studies were deemed of moderate or good quality. Sensitivity analyses demonstrated that none of the above issues substantially altered the overall effect size.

There were some potential sources of bias in this study. Three studies reported high rates of loss to follow-up (>20 %), which may have included a disproportionate number of unascertained deaths, especially if patients that died later were more likely to be LAM-negative. A sensitivity analysis showed that excluding studies with high loss to follow-up did not alter effect size. Many studies were excluded from the systematic review because they did not report mortality rates, and assessment for publication bias revealed a lack of smaller studies demonstrating small, weak associations or no association at all. Most studies reporting urinary LAM testing in HIV-TB co-infection were studies on diagnostic accuracy. It is likely that the majority of these did not gather mortality data, and studies included in this review mostly reported mortality as a secondary outcome. Finally, only five studies reported adjusted multivariable models for mortality that included urinary LAM detection as a variable, making exploration of heterogeneity by sub-group analysis challenging.

The mechanism by which LAM enters the urine has been an issue of contention, with early literature assuming LAM antigenuria resulted from free renal glomerular filtration of circulating LAM from the bloodstream [22, 23]. However, some researchers had also speculated that haematogenous dissemination of TB resulted in renal involvement and direct shedding into the urine, and this mechanism has now been demonstrated beyond doubt by multiple lines of evidence, including post-mortem data [24–27]. Moreover, urinary LAM has also been associated with mycobacteraemia and other markers of higher mycobacterial burden [19, 28–31]. Therefore, urinary LAM may simply be a marker of more severe, disseminated TB, explaining its association with mortality. Early studies of urinary LAM detection in patients with HIV-TB co-infection also demonstrated an association with TB immune reconstitution inflammatory syndrome (IRIS) [32], although this is unlikely to be an important a cause of increased mortality because TB-IRIS is rarely fatal [33].

Urinary LAM assays are also more sensitive in patients with lower CD4 cell counts (typically <100 cells/μl) [11, 25], because these patients have the highest risk of disseminated and renal TB. However, these are the very patients with the highest mortality risk [34], and therefore the higher mortality risk associated with urinary LAM detection may be confounded by CD4 cell count. However, of particular note, we have demonstrated in this meta-analysis that, even after adjusting for CD4 cell count as well as other predictors of mortality, urinary LAM remains an independent marker of mortality. Although mortality in LAM-negative TB cases is lower than that in LAM-positive cases, it was still up to 40 % in some studies. Without post-mortem data, it is difficult to attribute cause of death to TB or other co-infection or pathologies.

LAM itself may be on the causal pathway leading to increased mortality risk. Much in vitro research has examined the role of LAM as a virulence factor for *Mycobacterium tuberculosis* (*MTB*) [35]. LAM is a 19 kDa glycolipid and is a major constituent of *MTB* and the mycobacterial cell wall. It binds to several cell surface receptors of the immune system, especially macrophages [36, 37]. Through its immunomodulatory effects, LAM is thought to promote the survival of *MTB* in the human host [38] by directly impairing host immune defences. Its immunological effects include inhibition of cytokines that are key in the host immune response to *MTB*, such as interleukin (IL)-12, tissue necrosis factor alpha (TNF-α) and other inflammatory mediators [39–41]. Furthermore, LAM has been shown to enhance the secretion of anti-inflammatory cytokines such as IL-10 [42, 43]. LAM contributes to increased survival of *MTB* in macrophages, employing mechanisms that include prevention of macrophage phagosome maturation [44, 45]. These immunosuppressive effects of LAM could impair host responses to *MTB* and also to other opportunistic infections, and thereby contribute to increased mortality risk amongst patients who test positive for urinary LAM. In contrast, non-pathogenic mycobacteria contain structurally different LAM molecules to *MTB* that promote strong pro-inflammatory responses [38, 46].

The diagnostic utility of urinary LAM assays appears to be limited to HIV-positive patients with advanced immunosuppression. It is important to note that whilst we have found that patients with LAM detected in their urine had a higher mortality risk from the point of diagnosis and commencing anti-TB treatment, it is plausible that the use of urinary LAM assays to expedite the diagnosis and treatment of TB can also reduce mortality. This hypothesis and the impact of urinary LAM assays as a diagnostic tool in HIV-TB are currently being evaluated [14–16].

However, these assays identify patients at significantly increased risk of mortality during follow-up on TB treatment, above and beyond that accounted for by advanced immunosuppression and TB diagnosis. This appears to be true even in populations in which urinary LAM testing is not recommended for diagnostic purposes. This finding should prompt further research into whether interventions, in addition to the timely initiation of anti-TB therapy and ART, might benefit these patients and reduce their high mortality risk. Given the potential immunosuppressive nature of LAM itself, research is warranted to explore whether patients who are LAM-positive have altered immune responses compared to those who are LAM negative. Potential adjunctive interventions for these patients might be needed.

## Conclusions

This systematic review and meta-analysis has shown that patients with HIV-TB and detectable urinary LAM have increased mortality risk compared to those patients with TB without detectable urinary LAM. Urinary LAM is an independent risk factor for mortality, suggesting this finding is not simply an epiphenomenon detecting patients with more advanced immunosuppression. We have presented several plausible biological explanations for this association, including LAM’s immunosuppressive effects in vitro. Urinary LAM detection appears to be a feasible tool to highlight patients at high risk of mortality as well as identifying potential targets for adjunctive therapeutic interventions for reducing TB deaths over the next 20 years.

## Additional file

Additional file 1: Table S1. Search Strategy. **Table S2.** Quality assessment tool. **Table S3.** Additional information about studies included in the systematic review. **Table S4.** Effect estimates of mortality risk ratio for urine LAM-positive TB patients compared to urinary LAM-negative TB patients from sub-group analyses. **Figure S1.** Funnel plot of log odds ratios plotted against the standard error of the log odds ratio. (DOCX 25 kb)
