# Supplementary material for: Detection of lipoarabinomannan (LAM) in urine is an independent predictor of mortality risk in patients receiving treatment for HIV-associated tuberculosis in sub-Saharan Africa: a systematic review and meta-analysis
Source: BMC Med. 2016 Mar 23;14:53. doi: 10.1186/s12916-016-0603-9 (PMC4804532; doi:10.1186/s12916-016-0603-9)
Supplement: Additional file 1: Table S1. — Search Strategy. Table S2. Quality assessment tool. Table S3. Additional information about studies included in the systematic review. Table S4. Effect estimates of mortality risk ratio for urine LAM-positive TB patients compared to urinary LAM-negative TB patients from sub-group analyses. Figure S1. Funnel plot of log odds ratios plotted against the standard error of the log odds ratio. (DOCX 25 kb) [file 12916_2016_603_MOESM1_ESM.docx]

**Table S1: Search Strategy**

| Search concept | Search Terms |
| --- | --- |
| 1. HIV/AIDS | HIV-1* OR HIV-2* OR HIV* or HIV Infections* OR HIV Seropositivity* OR “human immunodeficiency“ OR  “human immune deficiency virus” OR human immune-deficiency syndrome OR human immune-deficiency syndrome AIDS OR “Acquired Immunodeficiency Syndrome”* OR acquired immune deficiency syndrome.mp. |
| 1. LAM | Lipoarabinomannan* OR LAM* |
| 1. Tuberculosis | TB* OR Tuberculosis* OR Mycobacterium tuberculosis* TUBERCULOSIS* |
| 1. Sub-Saharan Africa* | “Africa South of the Sahara"* OR Central Africa* OR Western Africa* OR Eastern Africa* OR Southern Africa* OR Benin* OR Benin* OR Burkina Faso* OR Burundi* OR Central African Republic* OR Chad* OR Comoros* OR “Democratic Republic of the Congo"* OR Eritrea* OR Ethiopia* OR Gambia* OR Guinea* OR Guinea-Bissau* OR Kenya* OR Liberia* OR Madagascar* OR Malawi* OR Mali* OR Mozambique* OR Niger* OR Rwanda* OR Sierra Leone* OR Somalia* OR Tanzania* OR Togo* OR Uganda* OR Zimbabwe* OR Cameroon* OR Cape Verde* OR Congo* OR Cote d'Ivoire* OR Ghana* OR Lesotho* OR Mauritania* OR Nigeria* OR Atlantic Islands* OR Senegal* OR Sudan* OR South Sudan* OR Swaziland* OR Zambia* OR Angola* OR Botswana* OR Gabon* OR Mauritius* OR Namibia* OR Seychelles* OR South Africa* OR Equatorial Guinea* |
|  | All the above sets (1-4) were combined with “AND” |

All terms were searched as keywords, * denotes also searched as subject heading word and MeSH,
$ denotes truncation, * based on OVID expert search strategy for ‘countries of sub-Saharan Africa’.

**Table S2: Quality assessment tool**

| **QUALITY ASSESSMENT TOOL** | | | |
| --- | --- | --- | --- |
| **Selection of study participants** | **Score** | | |
| Was the spectrum of patients representative of the patients who will undergo urine LAM testing? | 0 | 0.5 | 1 |
| Were selection criteria clearly described? | 0 | 0.5 | 1 |
| What was the HIV prevalence amongst TB-case (90 to <100%=0.5, 100%=1) | 0 | 0.5 | 1 |
| **Total** |  | | |
| **Testing** | **Score** | | |
| Is the reference standard for tuberculosis adequate? | 0 | 0.5 | 1 |
| Was the methodology for performing LAM testing adequate? | 0 | 0.5 | 1 |
| What cut-off was used for positive LAM test (not described/grade 1= 0.5, grade 2=1, ELISA=1)? | 0 | 0.5 | 1 |
| **Total** |  | | |
| **Mortality ascertainment** | **Score** | | |
| Was method of ascertaining mortality clearly described? | 0 | 0.5 | 1 |
| Was overall mortality rate appropriate for the clinical setting? | 0 | 0.5 | 1 |
| Was the loss-to-follow rate appropriate? | 0 | 0.5 | 1 |
| Was a multivariate risk factor analysis performed? | 0 | 0.5 | 1 |
| **Total** |  | | |
| **OVERALL TOTAL (out of 10)**  **<5= poor**  **5-7.4= moderate**  **>7.4=good** |  | | |

**Table S3: additional information about studies included in the systematic review**

| **Study** | **LAM assay used (type of urine sample)** | **LAM cut-off (based on pre-2014 TB-LAM reference card)** | **TB reference standard** | **Method of ascertaining mortality** | **Overall loss to follow-up (%)** | **Median CD4 cell count (cells/mm^3^)** | **Quality assessment score (<50 poor, 50-74 moderate, >74 good)** |
| --- | --- | --- | --- | --- | --- | --- | --- |
| **Balcha et al (2014)** | Determine TB-LAM lateral flow assay (frozen urine) | not reported | Positive TB culture or Xpert from sputum or lymph node aspirates | not reported | 3.6 | 176  (LAM +ve 94; LAM –ve 187) | 60 |
| **Lawn et al (2012)** | Determine TB-LAM lateral flow assay (frozen urine) | not reported | Positive TB culture from sputum | record review | 13.4 | 100  (LAM+ve 37; LAM-ve 115) | 70 |
| **Manabe et al** **(2014)** | Determine TB-LAM lateral flow assay (fresh urine) | any band | Positive TB culture from any specimen | record review and follow-up appointment | 21.1 | 57 | 75 |
| **Talbot et al (2012)** | Clearview TB ELISA (fresh and frozen urine) | OD >450nm | Positive TB culture from sputum or blood culture | follow-up, tracing | 43 | 86 | 75 |
| **Drain et al (2015)** | Determine TB-LAM lateral flow assay (frozen urine) | grade 1 | TB symptoms (63% culture-confirmed) | follow-up | 0 | 168  (LAM+ve 106; LAM–ve 198) | 70 |
| **Shah et al (2009)** | Clearview TB ELISA (frozen urine) | OD >450nm | Positive TB culture from any specimen, or acid fast bacilli/granuloma | follow-up | 0 | 79 | 60 |
| **Peter et al (2013)** | Determine TB-LAM lateral flow assay (frozen urine) | grade 2 | Positive TB culture from any specimen | not reported | 15.8 | 89  (LAM +ve 62; LAM –ve 180) | 80 |
| **Peter et al (2015)** | Determine TB-LAM lateral flow assay (frozen urine) | grade 2 | Positive TB culture from sputum | follow-up | 32.6 | 210 | 85 |
| **Lawn et al (2015)** | Determine TB-LAM lateral flow assay (frozen urine) | grade 2 | Positive TB culture or Xpert from any specimen | not reported | NR | 148 | 75 |
| **Bjerrum et al (2015)** | Determine TB-LAM lateral flow assay (fresh urine) | grade 2 | Positive TB culture or Xpert from sputum | record review, tracing | 13.4 | 127 | 70 |

**Table S4: Effect estimates of mortality risk ratio for urine LAM-positive TB patients compared to urine LAM-negative TB patients from sub-group analyses**

| **Sub-group** | **Number of studies included in analyses** | **Summary mortality risk ratio (95% CI)** | **Heterogeneity** |
| --- | --- | --- | --- |
| **Overall summary estimate** | **10** | **2.3 (1.6-3.1)** | **I^2^=37.0%** |
| Hospitalised patients | 6 | 1.9 (1.5-2.5) | I^2^= 0% |
| Outpatients | 4 | 3.4 (1.2-9.5) | I^2^= 69% |
| Overall TB mortality >20% | 5 | 1.8 (1.4-2.2) | I^2^= 0% |
| Overall TB mortality ≤20% | 5 | 3.7 (2.2-6.2) | I^2^= 0% |
| Median CD4 cell count ≤100 cells/μl | 5 | 1.9 (1.4-2.6) | I^2^= 12% |
| Median CD4 cell count >100 cells/μl | 5 | 2.7 (1.5-4.7) | I^2^= 52% |
| Mortality measured ≤3months | 6 | 2.1 (1.5-2.9) | I^2^= 21% |
| Mortality measured >3months | 4 | 2.6 (1.3-5.2) | I^2^= 61% |

All analyses using random-effects meta-analysis

**Figure S1: funnel plot of log odds ratios plotted against the standard error of the log odds ratio.**
